# A novel self-micro-emulsifying delivery system (SMEDS) formulation significantly improves the fasting absorption of EPA and DHA from a single dose of an omega-3 ethyl ester concentrate

**DOI:** 10.1186/s12944-017-0589-0

**Published:** 2017-10-16

**Authors:** Yan Qin, Hilde Nyheim, Else Marie Haram, Joseph M. Moritz, Svein Olaf Hustvedt

**Affiliations:** 1 Pronova Biopharma Norge AS, part of BASF, P.O. Box 420, NO-1327 Lysaker, Norway; 2 BASF Corporation, 100 Park Ave, Florham Park, NJ 07932 USA

**Keywords:** Omega-3-acid ethyl ester (EE), Eicosapentaenoic acid (EPA), Docosahexaenoic acid (DHA), Self-micro emulsifying delivery system (SMEDS), Absorption, Bioavailability

## Abstract

**Background:**

Absorption of EPA and DHA from Omega-3-acid ethyl ester (EE) concentrate supplements occurs most efficiently when taken in context of a fatty meal; adequate fat intake is required to release bile salts that emulsify and pancreatic enzymes that digest omega-3-containing lipids in the intestine. Current guidelines recommend reduction in fat intake and therefore there is a need to optimize the absorption of Omega-3 in those consuming low-fat or no-fat meals. To this end, BASF has developed an Absorption Acceleration Technology, a novel self-micro-emulsifying delivery system (SMEDS) formulation of highly concentrated Omega-3-acid EE which enables rapid emulsification and microdroplet formation upon entering the aqueous environment of the gut therefore enhances the absorption.

**Methods:**

Two separate single dose, crossover studies were conducted to determine the relative bioavailability of omega-3-acid EE concentrate, either as a novel SMEDS formulation (PRF-021) or as control, in healthy fasted male and female adults at two dose levels (Study 1 “low dose”: 630 mg EPA + DHA in PRF-021 vs. 840 mg EPA + DHA in control; Study 2 “high dose”: 1680 mg EPA + DHA in PRF-021 vs. 3360 mg EPA + DHA in control). Blood samples were collected immediately before supplementation and at defined time intervals for 48 h. Plasma concentration of total EPA and DHA were determined for pharmacokinetic analysis, area under the curve (AUC) and maximum observed concentration (C_max_) was determined.

**Results:**

Total EPA plus DHA absorption from SMEDS formulation PRF-021 were 6.4 and 11.5 times higher compared to control in low- and high-dose studies respectively, determined as the ratio of baseline corrected, dose normalized AUC_0-24h_ of PRF-021 over that of control. EPA and DHA individually showed differing levels of enhancement: the AUC_0-24h_ ratio for EPA was 23.8 and 25.7 in low and high dose studies, respectively, and the AUC_0-24h_ ratio for DHA was 3.6 and 5.6 in low and high dose studies, respectively. C_max_ was also increased for both EPA and DHA 2.7- to 9.2-fold.

**Conclusion:**

PRF-021 is a novel SMEDS formulation of Omega-3-acid EE demonstrating a marked improvement in absorption of a single dose of EPA and DHA EE under fasted conditions. This allows adequate absorption of Omega-3 from the supplement without the requirement of a high-fat meal.

## Background

Omega-3 fatty acids integrate into cell membranes of every cell type in the body, and supplemental Omega-3 lipids have been studied with respect to a variety of health promoting activities, in disparate bodily systems such as brain, eye, skin, musculoskeletal, joint, and cardiovascular. Delivery of Omega-3 fatty acids to each of these cell types requires adequate delivery to the bloodstream, pointing to the need to optimize their bioavailability.

For example, the importance of bioavailability is evident regarding supplementation of Omega-3 lipids for cardiovascular health. Strong epidemiologic evidence points to the cardiovascular health benefit of higher intake, blood levels, and cellular stores of Omega-3 fatty acids, specifically EPA and DHA [1–5]. Recent reviews and meta-analyses confirm the effectiveness of Omega-3 treatment in risk reduction of secondary cardiovascular outcomes [6–8]. Nevertheless, recent clinical trials have reached neutral conclusions with respect to primary cardiovascular outcomes in the use of Omega-3 supplementation in risk reduction of cardiovascular disease [9, 10]. One hypothesis attempting to explain this discrepancy is that Omega-3 supplement bioavailability was not optimized during study design, for example, due to the lack of recommendations to take supplements with a high-fat meal [4].

Optimal absorption of Omega-3 fatty acid supplements occur with intake of a fat-containing meal due to stimulation of bile release as reviewed by Schuchardt [11]. The impact of bile secretion is two-fold: 1) bile acids promote emulsification of the fats to microdroplets to allow adequate access to digestive enzymes, and 2) pancreatic digestive enzyme hydrolyze the fats to fatty acids and monoglycerides which are then available for intestinal absorption.

**Fig. 1:**
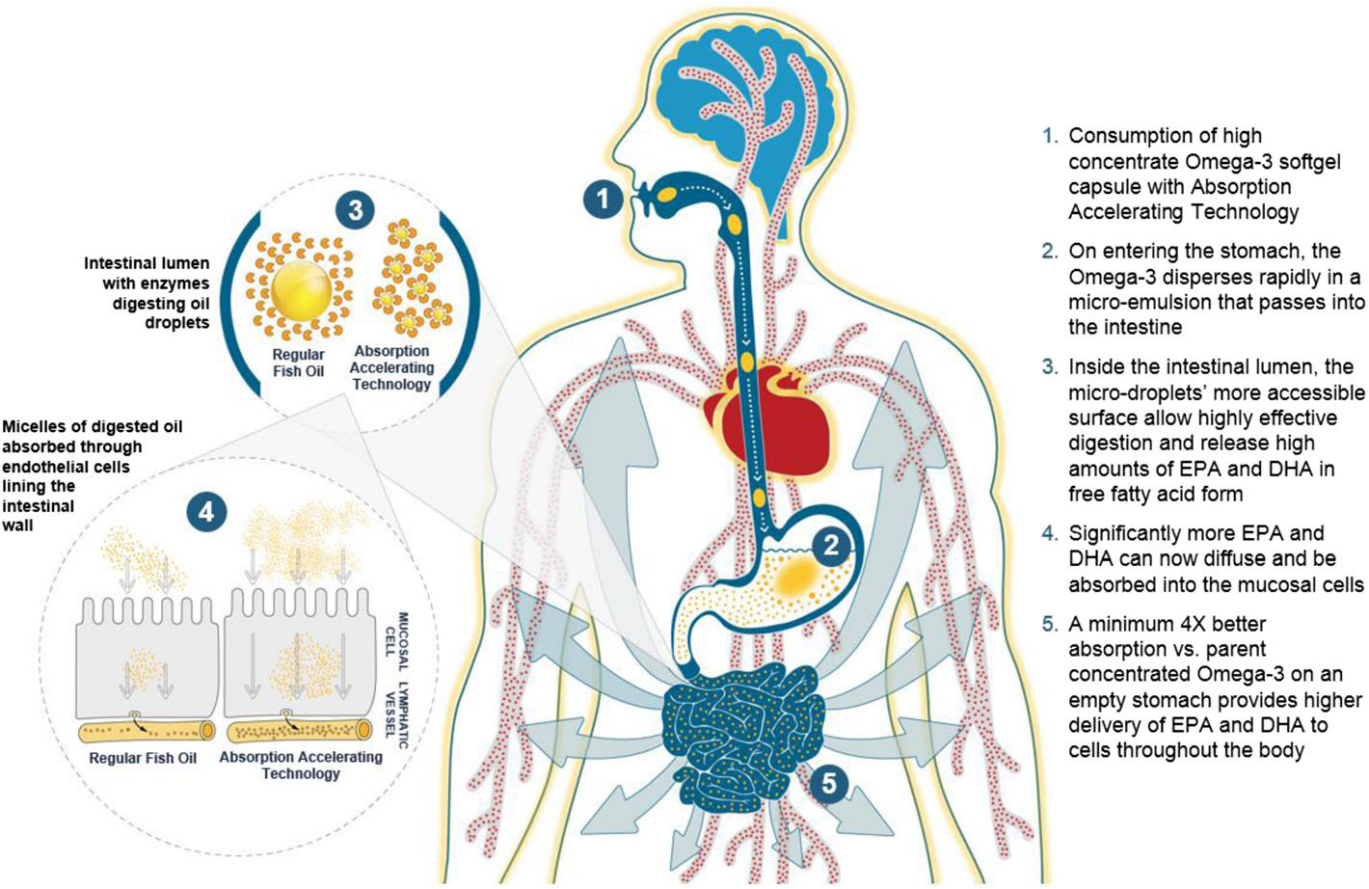
Absorption accelerating technology

It is not always desired or convenient to take supplements with a fatty meal, and in addition, those at risk of heart disease are counseled to reduce animal fat intake [12]. To promote greater absorption of Omega-3 without a high fat meal, it is possible to simulate the emulsification function of bile release by designing a self-micro-emulsifying delivery system (SMEDS). To this end, we have designed an Absorption Accelerating Technology: a proprietary SMEDS Omega-3 technology (specific formulation PRF-021) (Fig. 1) which is a liquid softgel containing highly concentrated Omega-3 ethyl esters, food-grade carrier oils, emulsifiers, and antioxidants which are spontaneously emulsified to microdroplets in the gastric contents.

The present paper presents the results of two studies designed to compare the bioavailability of a single dose of Omega-3 SMEDS formulation PRF-021 to its parent high concentrate Omega-3 ethyl ester oil at two dosage amounts in healthy, fasted subjects.

## Methods

### Study design and procedures

Two separate studies were conducted at Covance Clinical Research Unit (CRU). Both trials are open-label, randomized, cross-over single dose studies.

Study 1 was conducted in Allschwil, Switzerland and Study 2 in Leeds, U.K. Prior to the start of the study, the protocol and Informed Consent Form were reviewed and approved by the local Ethics Committee (EC). The study commenced after receipt of a EC approval. The regulatory permission to perform the study was obtained in accordance with applicable regulatory requirements. All ethical and regulatory approvals were available before a subject was exposed to any study-related procedure. The studies were conducted in accordance with Covance standards meeting requirements of Good Clinical Practice guidelines, the Declaration of Helsinki and local laws. Each subject was given a volunteer information, which provided details on the investigational products, procedures and potential risks involved and all subjects were given the option to obtain further information from the Investigator. A written informed consent was obtained from all subjects before they entered the study. Subjects were not permitted to take any medication, herbal products, omega-3 fatty acid supplements or fish oil preparations within 14 days before the first dose of the study. Subjects were instructed not to eat more than one fatty fish meal per week within this 14-day period. Vitamins and mineral supplements not containing omega-3 fatty acids or fish oil and non-containing other substances were allowed until 48 h before each dose.

### Participants

A total of 20 and 40 subjects were randomized in Study 1 and 2, respectively. One subject was withdrawn in the PRF-021 treatment in Study 2, so a total of 20 and 39 subjects finished in Study 1 and 2, respectively. In both trials, healthy adult subjects of any ethnic origin, aged between 18 and 55 years, with a body mass index (BMI) between 18.5 and 30.0 kg/m^2^, were selected. From 14 days before the administration of the first dose until completion of the post study visit, subjects were required to eat no more than 1 fish meal per week and to avoid taking fish oil or omega-3-fatty acids supplements. There were also limitations for intake of poppy seeds, Seville oranges, grapefruit and caffeine containing foods and beverages. Summary of main demographic data is listed in Table 1.

**Table 1:** Summary of screening demographic data in Study 1 and 2

	Study 1	Study 2
Overall	N = 20	N = 40
Age (years)	45 (24–55)	31 (19–55)
Male	17 (85.0%)	20 (50.0%)
Female	3 (15.0%)	20 (50.0%)
Race white	19 (95.0%)	38 (95.0%)
Race non-white	1 (5.0%)	2 (5.0%)
Ethnicity Hispanic or Latino	1 (5.0%)	1 (2.5%)
Not Hispanic or Latino	19 (95.0%)	39 (97.5%)
Weight (kg)	82.2 (56.3–98.6)	70.9 (47.3–105.8)
BMI (kg/m^2^)	26.8 (22.3–29.4)	23.5 (18.5–29.3)

Data presented as average (min-max), or participant numbers (percentage)

### Dosing

Each treatment period consisted of an in-clinic stay at the unit lasting from Day −1 (the evening before dosing) to Day 3 (48 h post-dose). On each dosing occasion, subjects fasted for at least 10 h overnight before dosing and approximately 4.5 h after dosing, when a standard low-fat (<15 g total fat content) lunch was given. Each subject swallowed the appropriate number of capsules with approximately 240 mL of water at room temperature. Subjects were not allowed any other intake of fluids from 1 h prior to until 2 h after dosing. Standard meals designed for bioavailability studies not containing fish were provided for all subjects whilst resident in the clinical research unit. In both studies, after an initial washout period, subjects were randomized to either a PRF-021 or control with a crossover washout period of at least 6 treatment-free days between each treatment. A crossover design was chosen in both studies to give a within-subject assessment of the absorption of EPA and DHA and to increase the power of the study for the given number of subjects. The evaluated omega-3 fatty acids EPA and DHA are dietary compounds for which quantifiable endogenous plasma levels are to be expected in healthy subjects. In prior studies at similar doses (data on file), the EPA and DHA levels were back to the baseline value by about 48 h after dosing. Hence, a washout period of at least 6 treatment-free days (the first treatment-free day started 24 h after the dosing time) between each study treatment is considered appropriate for practical reasons, and sufficient to avoid carry-over effects.

Omega-3 (EPA and DHA) content of each supplement group is given in Table 2. In Study 1, subjects were given two SMEDS-formulated PRF-021 capsules (total dosage: 630 mg EPA + DHA) or one control Omega-3 ethyl ester concentrate capsule (dosage: 840 mg EPA + DHA). In Study 2, subjects were given four SMEDS-formulated PRF-021 capsules (total dosage: 1680 mg EPA + DHA) or four control Omega-3 ethyl ester concentrate capsules (total dosage: 3360 mg EPA + DHA).

**Table 2:** Nominal EPA and DHA doses in Study 1 and 2

Study	Omega-3	PRF-021			Control		
		Per capsule (mg)	Capsules (#)	Per treatment (mg)	Per capsule (mg)	Capsules (#)	Per treatment (mg)
1. Low dose	EPA-EE	173		345	460		460
	DHA-EE	142	2	285	380	1	380
	EPA + DHA EE	315		630	840		840
2. High dose	EPA-EE	230		920	460		1840
	DHA-EE	190	4	760	380	4	1520
	EPA + DHA EE	420		1680	840		3360

PRF-021 features Absorption Acceleration Technology, which is a proprietary of ultra-high-concentration fish oil ethyl esters (>80% EPA + DHA) with food-grade excipients - carrier oils, antioxidants, and emulsifiers - designed to promote the spontaneous formation of micron-sized microdroplets in simulated gastric and intestinal fluids and optimized degradation by digestive enzymes (BASF internal data). An extensive number of different formulations were screened by the in vitro system thereafter a selected number were included in non-clinical bioavailability assessment. Bioavailability of the formulations were verified in a minipig absorption model (BASF internal data).

In Study 1, the lower dose administration range (less than 1000 mg of EPA + DHA) with lower variability was chosen to facilitate the comparison between PRF-021 and control within a dose range applicable for dietary supplement use. From a prior study (data not shown) where the control Omega-3 concentrate was administered as a 1000 mg single oral dose, the inter-individual coefficient of variance (CV%) of DHA, AUC and C_max_, were taken as a reference for the sample size calculation of this study. A sample size of 20 subjects (per treatment) was determined to provide the study a statistical power of 80% to demonstrate a difference in AUC in PRF-021 and control of more than 25% and an alpha level of 0.05. In the subsequent Study 2, the absorption comparison between PRF-021 and control was conducted at higher uptake level range (1000–4000 mg of sum EPA EE plus DHA EE) with expected higher variability, the sample size was increased to 40 individuals to achieve the above statistical analytical power.

### Blood samples collection and determination of EPA and DHA

In both studies, blood samples were drawn immediately before administration of the test product and at 0.25, 0.5, 0.75, 1, 2, 3, 4, 6, 8, 12, 16, 24 and 48 h’ post-dose. In Study 2, two additional blood samples were drawn at 1.5 and 36 h’ post-dose. Plasma were prepared within 2 h of blood withdrawal and stored at −80 °C until analysis.

The total EPA and DHA analysis in plasma samples were conducted in Study 1 by AS Vitas, Norway and in Study 2 by the Department of Bioanalysis and Immunology, Charles River Laboratories, Edinburgh UK. The samples were analyzed with validated chromatographic methods; gas chromatography with flame ionization detection (GC-FID) method in Study 1 and high performance liquid chromatography-mass/mass (HPLC-MS/MS) method in Study 2. The limit of quantification of total EPA and DHA in plasma is 5 μg/ml for both methods.

The EPA-EE and DHA-EE in plasma samples were analyzed with validated HPLC-MS/MS method. The limit of quantification of EPA-EE and DHA-EE is 20 ng/mL.

### Pharmacokinetic analyses

The pharmacokinetic parameters were determined using non-compartmental analysis from the plasma concentrations of EPA and DHA. The area under the plasma concentration-time curve (AUC) was calculated by applying the log-linear trapezoidal model to the measured EPA and DHA concentrations and the combined sum of EPA plus DHA at the actual sampling time points. In most data sets, it was not possible to define a terminal elimination phase. The elimination rate constant was therefore not calculated, and exposure is reported as the AUC from time 0 to 24 h post-dosing (AUC_0-24h_). The AUC_0-24h_ is chosen as it represents an intake interval of a daily intake of omega-3 supplement. C_max_ was defined as the maximum observed plasma concentration. Plasma concentration below the limit of quantification was set to zero.

The pharmacokinetic analysis has been made on EPA and DHA, both with pre-existing endogenous levels. Therefore, the data should be evaluated corrected for pre-existing baseline levels. The endogenous concentrations of EPA and DHA are variable and can therefore contribute significantly to the total amount of EPA and DHA and would add to the inter- and intra-individual variability. We therefore conducted a pre-dose adjustment of measured levels. For each subject and test period, the pre-dose concentrations were subtracted from the measured plasma concentrations and the PK parameters was calculated on the baseline-adjusted concentrations according to regulatory guidelines for bioequivalence testing of endogenous substances [13, 14]. If the measured value was lower than the pre-dose concentration the adjusted concentration was set to zero. Some subjects having a result of 0 in baseline-corrected AUC due to malabsorption over the 0–24 h time window and hence the baseline-corrected concentration values were zero or below zero for almost the entire time window. These individuals were excluded from the ratio calculations as their individual ratios could not be calculated. If the baseline level was missing, the subject was excluded from the PK analysis for that period.

### Statistical analyses

For both studies, the baseline corrected pharmacokinetic parameter data was provided to Covance CRU, Leeds, UK for further statistical analysis. The PK parameters were compiled including summary descriptive statistics (median, geometric mean with 90% confidence intervals (CI) and coefficient of variation (CV) arithmetic mean, standard deviation (SD) maximum and minimum values, and number of observations.

Statistical analysis was applied after dose normalization. The AUC_0-24h_, and C_max_ parameters were dose-normalized by division of the administered corresponding fatty acid in grams.

Least squares (LS) means were calculated for dose normalized AUC_0-24h_ and C_max_ parameters for both formulations. Mean differences between PRF-021 and control formulations were calculated. Individuals with AUC_0-24h_ levels below or equal to zero was excluded from the ratio calculations. The residual variance from the ANOVA was used to calculate 95% CI for the difference between treatments. These values were back-transformed to give geometric LS means, a point estimate and 95% CI for the ratio of PRF-021 to control.

All statistical analysis was pre-defined in the protocol or statistical plan for the studies.

### Adverse events

No serious or significant adverse events assigned to treatment were reported in either group for both studies. PRF-021 was well tolerated by healthy subjects during the study. One subject was withdrawn from the Study 2 due to an adverse event of gout which was deemed not related to study supplement.

## Results

Plasma concentrations of EPA plus DHA, and EPA and DHA individually, after single administration were significantly higher from PRF-021 vs. control despite lower doses of EPA and DHA.

**Fig. 2:**
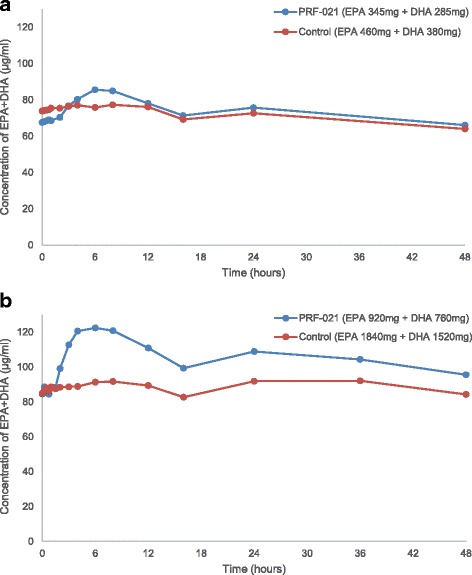
Sum of EPA + DHA concentration in plasma over time. Average value of sum of EPA + DHA concentration in plasma after a single oral dose of PRF-021 formulated Omega-3 EE or Control Omega-3 EE up to 48 h. (**a**) low dose Study 1, *N* = 20 (**b**) high dose Study 2, *N* = 39 for PRF-021 and *N* = 40 for Control

Plasma concentrations of EPA and DHA following single oral administrations of either PRF-021 or control were measured with respect to two dosage levels in fasting subjects (Fig. 2). The endogenous baseline concentrations of plasma EPA and DHA are shown in Table 3.

**Table 3:** Baseline plasma EPA and DHA concentration

Study		PRF-021 group mean ± SD (μg/mL)	Control group mean ± SD (μg/mL)
1. Low dose	EPA	21.5 ± 11.2 (N = 20)	25.8 ± 14.6 (N = 20)
	DHA	46.1 ± 16.7 (N = 20)	48.1 ± 15.2 (N = 20)
2. High dose	EPA	23.6 ± 10.4 (N = 39^a^)	23.7 ± 9.79 (N = 40)
	DHA	60.9 ± 18.4 (N = 39^a^)	61.2 ± 19.8 (N = 40)

^a^One subject withdrawn

There were no statistically significant differences in the baseline EPA and DHA levels between the two treatment groups in both studies. Because the endogenous levels of EPA and DHA significantly influence the assessment of systemic exposure, the pre-dose concentrations of EPA and DHA were subtracted on an individual basis, and the PK analysis was performed on baseline-corrected data.

**Fig. 3:**
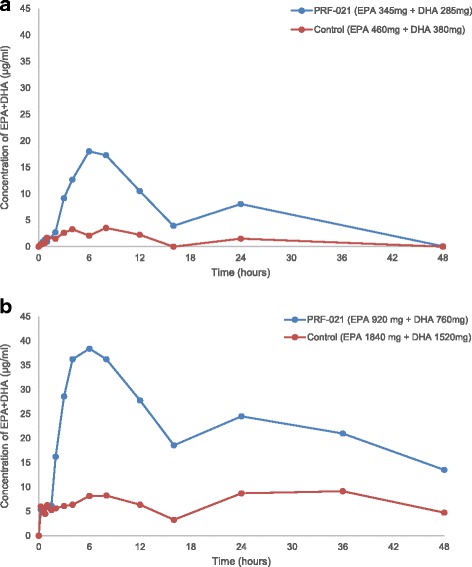
Baseline-corrected sum of EPA + DHA concentration in plasma over time. Average value of baseline-corrected sum of EPA + DHA concentration in plasma after a single oral dose of PRF-021 formulated Omega-3 EE or Control Omega-3 EE up to 48 h. (**a**) low dose Study 1, N = 20 (**b**) high dose Study 2, N = 39 for PRF-021 and N = 40 for Control

In Study 1, EPA and DHA in PRF-021 were given at lower dose levels, corresponding to those typically given by dietary supplement intake. Twenty individuals were given 840 mg of control EPA + DHA EE (1 g of control oil) and 630 mg of PRF-021 SMEDS formulated EPA + DHA EE (1.5 g of SMEDS formulation) in a cross-over design. Baseline corrected plasma concentration vs. time plots demonstrate that both EPA and DHA were significantly (*p* < 0.05) better absorbed by PRF-021 formulation vs. control (Fig. 3a), despite the absolute dosage of EPA + DHA in PRF-021 was only 75% of that of control.

In the subsequent Study 2, the comparison was conducted at a higher dose range (1000–4000 mg of omega-3-acid EE). Forty participants were given 3360 mg of EPA plus DHA EE in control (4 g of control oil) and 1680 mg of EPA plus DHA EE in PRF-021 (4 g of SMEDS formulation) in a cross-over design. Though the latter had only 50% of the EPA + DHA dosage vs. the control, the concentration-time plots clearly showed much higher plasma concentrations versus that from control (Fig. 3b).

### Dose-corrected pharmacokinetic parameters show 6- to 11-fold improvement in AUC_0-24h_ dependent upon dose

Baseline corrected dose-normalized total EPA plus DHA exposure parameters are presented in Table 4. In Study 1, the formulated omega-3-acid EE had approximately 6-fold higher AUC_0-24h_, determined as geometric least square means after dose normalization. In study 2, the dose normalized difference between formulated and reference omega-3-acid EE oil was even bigger, 11 times for AUC_0-24h_.

**Table 4:** Baseline-corrected, dose normalized total EPA plus DHA AUC_0-24h_

Study	Treatment	Arithmetic mean ± SD of AUC_0-24h_/D	Geometric mean of AUC_0-24h_/D	Ratio of dose normalized geometric mean of AUC_0-24h_/D^c^			
		μg*h/mL/g (N)	μg*h/mL/g (N)	N	Ratio PRF-021: Control	Lower 95% CI	Upper 95% CI
1	PRF-021	368.3 ± 156.4 (20)	331 (20)	19	6.2	4.3	9.0
	Control	75.30 ± 57.50 (19^b^)	53.2 (19^b^)				
2	PRF-021	358.3 ± 141.3 (39^a^)	331 (39^a^)	35	9.6	7.0	13.1
	Control	46.96 ± 31.05 (36^b^)	35.7 (36^b^)				

^a^One subject withdrawn ^b^Subject having a result of 0 was excluded ^c^Ratios calculated in subjects with complete dataset only

### Both EPA and DHA absorption are increased by PRF-021 – The relative improvement is more pronounced with EPA

**Fig. 4:**
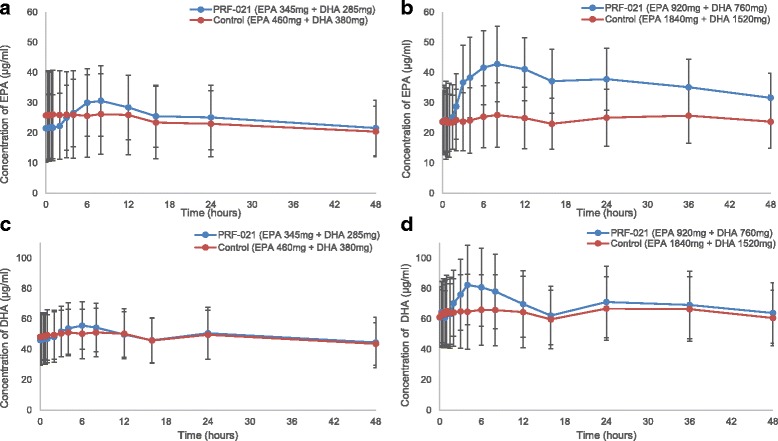
EPA and DHA concentration in plasma over time. Average value of EPA and DHA concentration in plasma after a single oral dose of PRF-021 formulated Omega-3 EE or control Omega-3 EE up to 48 h with standard error bar (**a**) low dose Study 1, EPA, N = 20; (**b**) high dose Study 2, EPA, N = 39 for PRF-021 and N = 40 for Control; (**c**) low dose Study 1, DHA, N = 20 and (**d**) high dose Study 2, DHA, N = 39 for PRF-021 and N = 40 for Control

**Fig. 5:**
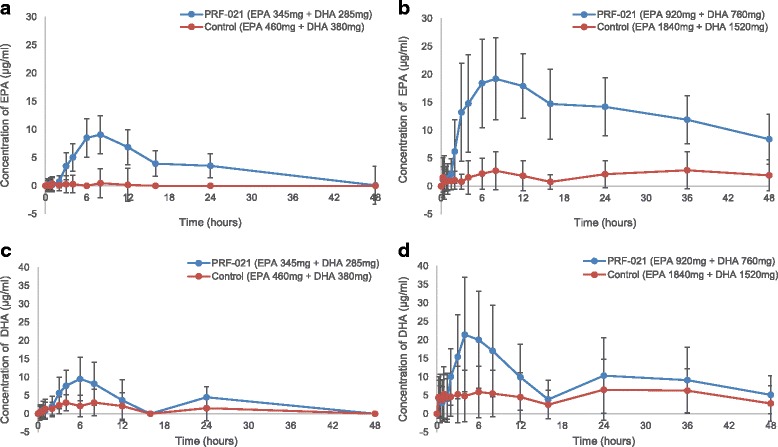
Baseline-corrected EPA and DHA concentration in plasma over time. Average value of baseline-corrected EPA and DHA concentration in plasma after a single oral dose of PRF-021 formulated Omega-3 EE or control Omega-3 EE up to 48 h with standard error bar (**a**) low dose Study 1, EPA, N = 20; (**b**) high dose Study 2, EPA, N = 39 for PRF-021 and N = 40 for Control; (**c**) low dose Study 1, DHA, N = 20 and (**d**) high dose Study 2, DHA, N = 39 for PRF-021 and N = 40 for Control

The absorption of EPA and DHA were also investigated individually (Fig. 4a-d). Plasma concentration of baseline corrected EPA or DHA concentrations vs. time data was plotted for both studies (Fig. 5a-d). After 1.5–2 h lag time, the EPA and DHA were absorbed into plasma reaching a maximum concentration at 4 and 8 h post dose. Plasma concentrations of individual fatty acids EPA (Fig. 5a and b) and DHA (Fig. 5c and d) were enhanced by PRF-021 vs. control despite lower dosing.

Individual baseline-corrected dose-normalized EPA and DHA exposure pharmacokinetic parameters were presented in Tables 5 and 6, respectively. For EPA, PRF-021 exhibited a 7-fold higher C_max_ and 24-fold higher AUC_0-24h_ vs. control in Study 1 and a 9-fold higher C_max_ and 26-fold higher AUC_0-24h_ vs. control in Study 2. For DHA, C_max_ was approximately 2.7-fold higher and AUC_0-24h_ was 3.6-fold higher vs. control in Study 1 and C_max_ and AUC_0-24h_ were increased more by 4.3 and 5.6 times, respectively vs. control in Study 2. The relative absorption improvement is more pronounced for EPA than for DHA in both studies.

**Table 5:** Baseline-corrected dose normalized total EPA AUC_0-24h_ and C_max_

Study	Treatment	Dose normalized parameter	Arithmetic Mean ± SD (N)	Geometric mean (N)	Ratio of dose normalized geometric mean^c^			
					N	Ratio PRF-021: Control	Lower 95% CI	Upper 95% CI
1	PRF-021	AUC_0-24h_/D μg*h/mL/g	355.9 ± 135.4 (20)	326 (20)	19	23.6	12.5	44.5
	Control		33.4 ± 33.4 (19^b^)	13.7 (19^b^)				
	PRF-021	C_max_/D μg/mL/g	26.3 ± 9.8 (20)	25.9 (20)	19	6.7	5.0	9.0
	Control		1.02 ± 5.6 (19^b^)	3.9 (19^b^)				
2	PRF-021	AUC_0-24h_/D μg*h/mL/g	382.6 ± 122.6 (39^a^)	357 (39^a^)	35	25.58	15.9	41.1
	Control		22.9 ± 20.1 (36^b^)	14.0 (36^b^)				
	PRF-021	C_max_/D μg/mL/g	20.9 ± 8.0 (39^a^)	24.5 (39^a^)	36	9.2	7.1	11.9
	Control		1.5 ± 1.8 (40)	2.7 (40)				

^a^One subject withdrawn; ^b^Subjects having a result of 0 was excluded ^c^Ratios calculated in subjects with complete dataset only

**Table 6:** Baseline-corrected dose normalized total DHA AUC_0-24h_ and C_max_

Study	Treatment	Dose normalized parameter	Arithmetic mean ± SD (N)	Geometric mean (N)	Ratio of dose normalized geometric mean^b^			
					N	Ratio PRF-021: Control	Lower 95% CI	Upper 95% CI
1	PRF-021	AUC_0-24h_/D μg*h/mL/g	383.2 ± 202.8 (20)	325 (20)	20	3.63	2.57	5.14
	Control		122.2 ± 93.10 (20)	89.5 (20)				
	PRF-021	C_max_/D μg/mL/g	33.4 ± 20.9 (20)	34.3 (20)	20	2.68	2.09	3.44
	Control		7.95 ± 5.68 (20)	12.8 (20)				
2	PRF-021	AUC_0-24h_/D μg*h/mL/g	328.9 ± 195.1 (39^a^)	266 (39)	39	5.61	3.97	7.92
	Control		70.70 ± 53.88 (40)	48.1 (40)				
	PRF-021	C_max_/D μg/mL/g	28.2 ± 20.4 (39^a^)	36.4 (39^a^)	39	4.29	3.31	5.55
	Control		3.88 ± 4.56 (40)	8.50 (40)				

^a^One subject withdrawn ^b^Ratios calculated in subjects with complete dataset only

## Discussion

The current studies demonstrate that the application of Absorption Accelerating Technology a novel proprietary SMEDS technology, results in an increase of more than 6-fold in absorption as measured by ratios of baseline-adjusted, dose-normalized AUC_0-24h_ of a single dietary supplement dose of total EPA + DHA in ethyl ester high concentrate in healthy fasting adults. These studies were designed to determine if absorption of EPA and DHA can be improved under a potential lowest-absorption scenario: Omega-3 ethyl ester consumed during fasting conditions. This report corroborates the result of a recent study which demonstrated a similar improvement in bioavailability from a micro-emulsified Omega-3 EE [15]. In contrast to Lopez-Toledano [15], this report demonstrated a quantitatively greater improvement in bioavailability due to fasted feeding conditions (the prior study was carried out with consumption of a low-fat meal – therefore inducing greater absorption of EE control). In this case, also the absorption improvement was verified for both a low and a high dose (with the lower dose targeting dietary supplement consumption) whereas the prior study featured a single, higher dose.

In this report the relative improvement of bioavailability is greater for EPA than for DHA. This result corresponds to a previous trial demonstrating greater absorption of single doses of Omega-3 ethyl esters under similar condition as the current study [16].

The relative bioavailability improvement for total EPA + DHA was greater under high dosage conditions (10-fold) versus low dosage (6-fold). This effect was primarily due to a relative change in absorption of DHA between the low and high dose studies (AUC_0-24h_ ratio 3.6 and 5.6 respectively), whereas the ratio for EPA did not significantly change between the low and high dose cases. As the dose-adjusted AUC_0-24h_ of DHA between the low-dose and high dose studies for PRF-021 are quantitatively similar, the difference in AUC ratio points to a difference in the dose-normalized DHA absorption of the control EE oils between low and high dose. This is indicative of a lower per mg absorption at higher doses for DHA (and thus a lower number in the denominator of the AUC ratio) due to limited digestive enzyme and emulsification activity in the fasted gut.

SMEDS-formulated Omega-3 EE utilizes a softgel containing a commonly available cost-effective Omega-3 ethyl ester format and has various advantages over current approaches to improve bioavailability. Ready-made oil-in-water emulsions formats increase single-dose absorption by factors of 1.3–1.5 over standard triglyceride fish oils [17, 18] but may have issues with oxidative stability and require liquid packet or spoonable delivery formats. Krill oil demonstrated a slight single dose absorption improvement of approximately 1.5X of standard fish oil [19, 20], but still require hydrolyzation by gut enzymes, in addition, the cost to gain clinically relevant EPA + DHA doses is significantly higher than fish oil-derived ethyl esters. Standard fish oil triglycerides have greater absorption of a single dose versus ethyl esters with a low-fat meal by a factor of approximately 3 [21] but also require consumption with meals as absorption is still limited by emulsification and digestion in the gut [22]. Omega-3 free-fatty acids (FFA)‘s demonstrated improved single-dose absorption in low-fat fed conditions over Omega-3 ethyl esters by up to a factor of 4 [16], however FFA’s are currently only widely available by prescription and require specialized formulation to reduce negative gastrointestinal effects of free fatty acids [16].

As this technology demonstrates bioavailability of Omega-3 in the condition of lowest expected absorption, at fasted stomach, it can be expected that bioavailability is assured regardless of stomach content. A recent study demonstrated that emulsified Omega-3 showed similar bioavailability in both low and high fat fed conditions [22]. This has potential benefit for those wishing to take supplements at early morning and late night occasions. Dietary surveys show that Americans consume only 16% of total fat in the morning for breakfast [23] – this means that for a large segment of the population breakfast is considered a “low fat” meal (<10 g fat). Average transit time of gastric emptying time is approximately 1–2 h [24], and as such, those that take dietary supplements at night before bed might also represent consumption on a nearly empty stomach. This technology is potentially beneficial to those consuming low-fat diets for health, age or medical reasons; for instance, those wishing to reduce caloric consumption, improve blood triglyceride levels, or improve risk of heart disease [25].

A key limitation of this study is the single-dose design at different dose levels. To conclusively verify a potential bioavailability benefit of this technology, further long-term studies are needed analysing the integration of EPA + DHA into tissue fatty acid content, eg. in erythrocytes [13, 26].

## Conclusions

In conclusion, data from the two clinical studies demonstrate that the absorption of EPA + DHA EE in the SMEDS formulation was at least 6 greater than that of the reference omega-3-acid EE oil, determined by normalized AUC per gram of dose delivered. Individually, absorption increased at least by a factor of 23.6 for EPA and a factor of 3.6 for DHA. It is apparent that the absorption of Omega-3 EE is low in the fasted state and imperative that these supplements be taken with a fatty meal for appropriate absorption. Results from these two studies provide evidence of predictable increased absorption of EPA and DHA from the formulated product across the included intake range, without the need for a fatty meal.

## Abbreviations

AUC: Area under the curve
BMI: Body mass index
CI: Confidence interval
C_max_: Maximum observed plasma concentration
CV: Coefficient of variance
D: Dose
DHA: Docosahexaenoic acid
EC: Ethics Committee
EE: Ethyl ester
EPA: Eicosapentaenoic acid
MHRA: Medical and Healthcare products Regulatory Agency
SD: Standard deviation
SMEDS: Self micro emulsifying delivery system
